# A rare case of *Gardnerella vaginalis* spondylodiscitis

**DOI:** 10.1016/j.idcr.2024.e02126

**Published:** 2024-12-12

**Authors:** Alex Belote, Kassem Hammoud

**Affiliations:** Division of Infectious Diseases, Department of Medicine, University of Kansas, Kanas City, KS, USA

**Keywords:** Anaerobes, Gardnerella, *Gardnerella vaginalis*, Osteomyelitis, *Spondylodiscitis*, Discitis

## Abstract

A 55-year-old-male with a chronic left uretero-pelvic junction (UPJ) obstruction managed with intermittent stent exchanges presented with low midline back pain. CT Abdomen/Pelvis revealed spondylodiscitis at L4-L5, further demonstrated on MRI Lumbar spine. Imaging also revealed the left nephro-ureteral stent was mispositioned, with some mild wall thickening of the left ureter. He was not systemically ill, and antimicrobials were held. He underwent a L4/5 disc biopsy, and pathology revealed acute discitis. Blood and biopsy cultures remained negative through hospital day 5. He then underwent repeat L4/5 disc biopsy. Cultures of repeat biopsy resulted in *Gardnerella vaginalis*. IV antimicrobials were stopped, and oral Metronidazole was started. He completed 10 weeks of Metronidazole therapy, with significant clinical improvement.

*G. vaginalis* is a rare cause of bone and joint infections. It is difficult to culture and is less virulent than common bacteria associated with native vertebral osteomyelitis. There have been few case reports of *G. vaginalis* osteomyelitis or prosthetic joint infection, especially in males. *G. vaginalis* can rarely colonize the urethra in men and has been known to form biofilm on foreign material in the female genitourinary system. We suspect our patient had developed colonization of his ureteral stent, predisposing him to osteomyelitis. Were repeat biopsy not pursued in this case, our patient likely could have developed empiric treatment failure. Holding antibiotics after initial biopsy proved highly beneficial.

## Introduction

The differential diagnosis for pathogens in osteomyelitis varies depending on anatomic location and host risk factors. Vertebral osteomyelitis classically stems through hematogenous spread from a distant primary source [1]. The etiology for vertebral osteomyelitis is typically gram-positive pathogens, regardless of age, gender, or level of spinal involvement. In males younger than 60, *Staphylococcus*, *Streptococcus*, and *Enterococcus* combine to cause 86 % of cases [2], with *Staph. aureus* accounting for most of these cases [2], [3]. Less than 2 % of cases are due to anaerobes [2]. We present a rarely encountered culprit of osteomyelitis.

## Case

A 55-year-old-male with a chronic left uretero-pelvic junction (UPJ) obstruction presented to the ED in 2023 with complaints of 2–3 weeks of low back pain. He reported it was sudden onset, and there was no mechanism of injury. It had been gradually worsening over that timeframe. He described it as midline, sharp, 9/10 stabbing pain with radiation into bilateral hips. It worsened with movement and lying flat in bed. He experienced minimal relief with ibuprofen and started seeing a chiropractor without notable improvement. He missed a week of work related to these complaints.

He denied any systemic complaints, including fevers/chills, night sweats, other joint pain, bowel/bladder incontinence, or lower extremity weakness. He reported some blood-tinged urine recently but denied dysuria. He had a urinary tract infection about a year prior and has never required IV antibiotics.

He was known to have a UPJ obstruction since his 20 s, when it was incidentally identified during trauma work-up after he was involved in a car accident. He did not have any intra-abdominal or thoracic injuries related to this accident. Since that time, he had this UPJ obstruction managed with chronic stent exchanges. He did undergo two endoscopic balloon dilation procedures for this, one in the late 1990 's and one in the early 2000's, neither of which were successful. Recently he was evaluated by urology, and it was recommended that the patient undergoes left nephrectomy due to nonfunctional left kidney after a kidney scan was done. Surgery had not yet been performed. He has had several urinary tract infections throughout his adult life, for which he was treated with oral antibiotics. He has not required any antibiotics within the last year.

Upon presentation, the patient appeared well. His temperature was 98.8°F (37.1°C). He was normotensive with heart rates in the 80 s and maintaining oxygen saturations > 90 % on room air. Palpation of the cervical, thoracic, and lumbar spine showed no major tenderness on palpation. On exam the patient had difficulty moving around and sitting due to the low back pain. His exam was otherwise normal.

His white blood cell count was 11.2 k/µL (reference range 4.5–11.0) with 60 % neutrophils (normal 41–77 %). CRP was 3.88 mg/dL (reference range <1.00), and ESR was 43 mm/hour (reference range 0–20). Urinalysis was nitrite negative and had 3 + leukocytes (reference negative). There were 2–10 white blood cells/hpf seen on microscopy (reference 0–2), in the setting of 0–2 squamous epithelial cells/hpf (reference 0–5).

**Fig. 1:**
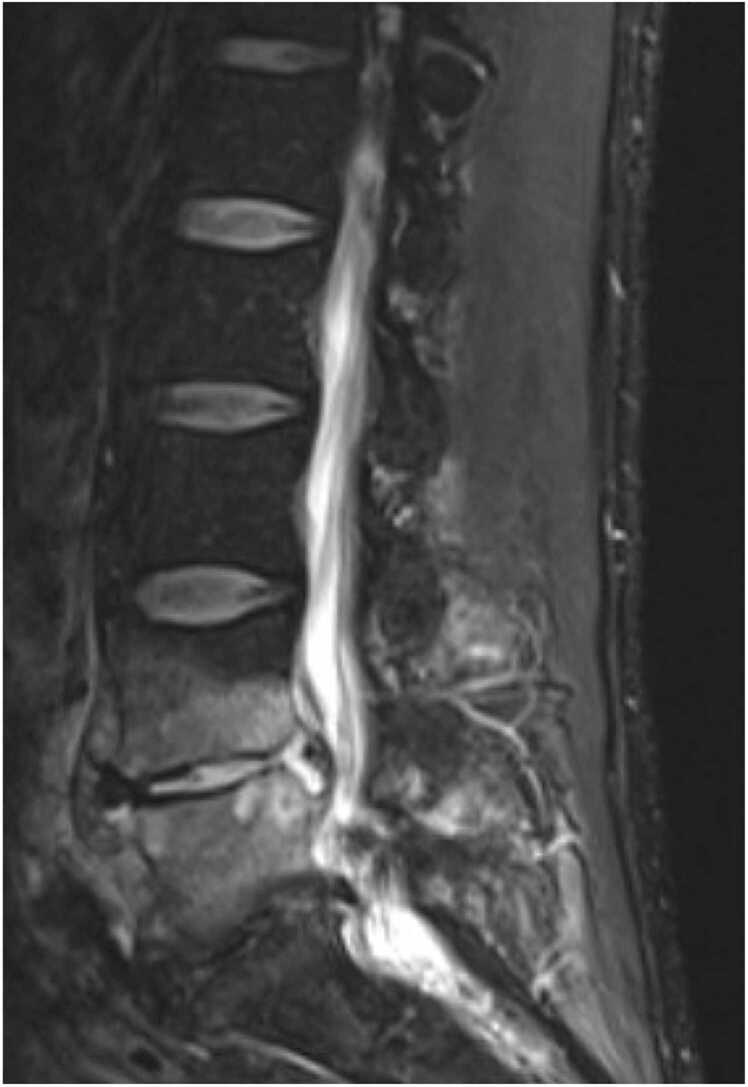
MRI Lumbar Spine.

Urine cultures and blood cultures were obtained. Given overall clinical stability, he was monitored off antimicrobials. CT abdomen/pelvis was obtained and revealed the development of cortical irregularity and osteolysis involving the L4–5 endplates and posterior superior aspect of the L5 vertebral body. There was also paravertebral edema about the L4 and L5 levels with new intermediate density material posterior to the L5 vertebral body, which resulted in moderate central canal narrowing. It was also noted that the left nephro-ureteral stent was mispositioned with the superior cope loop in the mid ureter, with some mild wall thickening of the proximal left ureter. MRI of the Lumbar Spine (Fig. 1) revealed loss of normal disc height with an abnormal fluid signal and diffuse contrast enhancement of the majority of the L4 and L5 vertebral bodies. There was enhancement in the epidural space, likely contiguous inflammation. Findings were compatible with spondylodiscitis at L4-L5 with contiguous phlegmon into the epidural space.

Urine culture ultimately resulted with < 100k CFU/mL urogenital/skin microbiota. On day 2 of admission, the patient underwent a L4/5 disc biopsy in interventional radiology. Pathology revealed focal necrosis and acute inflammation (discitis) with adjacent granulation tissue. AFB stains were negative for acid-fast bacilli, and GMS stains were negative for fungal organisms. Biopsy cultures remained negative on day 5 of admission. Two sets of peripheral blood cultures were also negative at 5 days. Transthoracic echocardiogram was obtained and was negative for vegetations.

On day 5, he underwent a repeat L4/5-disc biopsy. Results of repeat L4/5 disc biopsy were pending at the time of discharge on hospital day 5. He was started on empiric IV Daptomycin and IV Cefepime. The biopsy culture resulted with *Gardnerella vaginalis* on both routine and anaerobic cultures 12 days after initial admission. At that time, IV Daptomycin and IV Cefepime were stopped. Alternatively, oral Metronidazole 500 mg three times daily was started. He completed 10 weeks of Metronidazole therapy, with significant improvement in his pain. He ultimately underwent left nephrectomy 3 months following this and had no re-admissions since.

## Discussion

To our best knowledge, we present the first case of *G. vaginalis* vertebral osteomyelitis in a male. *G. vaginalis* is a rare cause of bone and joint infections. On average, anaerobes account for < 2 % of cases of vertebral osteomyelitis [2], and < 1 % of osteomyelitis in general [4]. Furthermore, *G. vaginalis* is even less common, particularly in males [5]. *G. vaginalis*’ main association is with bacterial vaginosis [6]. Several case reports to-date of *G. vaginalis* bone and joint infections have occurred in concurrence with *G. vaginalis* colonization of the genitourinary tract [7], [8].

Table 1 outlines all identified cases of *G. vaginalis* bone and joint infections in literature. To date, there have only been 3 cases of vertebral osteomyelitis reports in females [3], [9], [10], 1 case of native-joint septic arthritis in a female [11], 3 cases of prosthetic joint infections in females [5], [6], [8], 1 case of prosthetic joint infection in a male [5], and 1 case of skull osteomyelitis in a male infant whose gestation was complicated by bacterial vaginosis [7]. All cases of joint infections have been in the hip, and all adult cases of vertebral osteomyelitis in adults have been in the lumbar spine, suggesting a possible anatomic association with the GU tract. A variety of mechanisms have been proposed for genitourinary colonization/infection leading to lumbar spondylodiscitis, including hematogenous spread through the posterior venous plexus or Baston’s plexus [9], [10].

**Table 1:** Case reports of *Gardnerella Vaginalis* bone/joint infections in literature.

**Reference**	**Reference #**	Year*	**Age**	**Gender**	**Lesion**	**GV colonization**	**Polymicrobial**	**Surgical Management**	Antibiotic Therapy**	**Outcome**
Nightingale et al.	7	1986	Infant	M	Parietal OM	Yes (in mother)	No	Debridement	Ampicillin	Cure
Hodge et al.	9	1995	50	F	Vertebral OM	Unknown	No	No	Ampicillin	Cure
Graham et al.	10	2009	38	F	Vertebral OM	No	No	No	Clindamycin	Cure
Sivadon-Tardy et al.	11	2009	48	F	Hip Septic Arthritis	No	Yes	Debridement	Amoxicillin	Cure
Hoarau et al.	6	2012	71	F	Hip PJI	Unknown	Yes	1-stage revision	TMP-SMX	Cure
Thomas et al.	8	2019	68	F	Hip PJI	Yes	No	1-stage revision	Amoxicillin	Cure
Thomas et al.	8	2019	32	F	Hip PJI	Yes	No	1-stage revision	Clindamycin	Cure
Kim et al.	3	2021	61	F	Vertebral OM	No	Yes	No	Metronidazole	Cure
Saricaoglu et al.	5	2022	45	M	Hip PJI	No	Yes	Debridement/retention	Clindamycin	Cure
Belote et al.	present case	2024	55	M	Vertebral OM	Unknown	No	No	Metronidazole	Cure

M: Male

F: Female

OM: Osteomyelitis

GV: Gardnerella vaginalis

PJI: Prosthetic Joint Infection

* Year of publication

** Antibiotic used for main treatment course

*G. vaginalis* is a small, facultative anaerobic gram-variable rod [8], [9]. It is difficult to identify, difficult to culture, and less virulent [5], [7], [8] than more common bacteria associated with native vertebral osteomyelitis. *G. vaginalis* can form biofilms [6], [8], including on contraceptive intravaginal ring, but there is no data on extra-vaginal foreign bodies, or in males [5]. It can colonize the urethra in up to 4.5–11.4 % of males [5], and has been shown to colonize extravaginal mucosa in women [12]. We suspect that our patient had developed colonization of his left ureteral stent, given the noted wall thickening of the adjacent ureter on CT scan. It also may have been the “urogenital flora” grown on urine culture. Unfortunately, this specimen was not available for further analysis as his biopsy culture resulted 12 days after his original urine culture, and routine urine samples at our institution are discarded in that timeframe. We suspect that colonization of his ureteral stent with *G. vaginalis* is likely what predisposed him to develop native vertebral osteomyelitis. He improved on culture directed therapy, suggesting that the identified *G. vaginalis* was truly pathogenic.

Our case also highlights the diagnostic importance of withholding antibiotics for osteomyelitis in the setting of clinical stability, as per Infectious Disease Society of America (IDSA) Guidelines [1]. Biopsy results should guide therapy. Image-guided biopsy for lumbar spondylodiscitis has variable sensitivity, with reports as low as 40 % [13]. In the setting of a negative initial biopsy, IDSA guidelines recommend a 2nd biopsy to be sent for culture [1], as was performed in this case. Were repeat biopsy not pursued in this case, he likely would have developed empiric treatment failure and developed worsening symptoms, and potentially hospital re-admission. Holding antibiotics after initial biopsy in this case proved highly beneficial.

## CRediT authorship contribution statement

**Alex Belote:** Investigation, Writing – original draft. **Kassem Hammoud:** Conceptualization, Data curation, Investigation, Writing – review & editing.

## Declaration of Competing Interest

The authors declare the following financial interests/personal relationships which may be considered as potential competing interests: co-author serves as an editor for the journal ID Cases. KH If there are other authors, they declare that they have no known competing financial interests or personal relationships that could have appeared to influence the work reported in this paper.
